# Influence of Heat Treatment on Microstructure and Properties of NiTi46 Alloy Consolidated by Spark Plasma Sintering

**DOI:** 10.3390/ma12244075

**Published:** 2019-12-06

**Authors:** Pavel Salvetr, Jaromír Dlouhý, Andrea Školáková, Filip Průša, Pavel Novák, Miroslav Karlík, Petr Haušild

**Affiliations:** 1 COMTES FHT, Prumyslova 995, 334 41 Dobrany, Czech Republic; jaromir.dlouhy@comtesfht.cz; 2 Department of Metals and Corrosion Engineering, University of Chemistry and Technology, Technická 5, 166 28 Prague 6, Czech Republic; skolakoa@vscht.cz (A.Š.); prusaf@vscht.cz (F.P.); panovak@vscht.cz (P.N.); 3 Faculty of Nuclear Sciences and Physical Engineering, Department of Materials, Czech Technical University in Prague, Trojanova 13, 120 00 Prague 2, Czech Republic; miroslav.karlik@fjfi.cvut.cz (M.K.); petr.hausild@fjfi.cvut.cz (P.H.); 4 Faculty of Mathematics and Physics, Department of Physics of Materials, Charles University, Ke Karlovu 5, 121 16 Prague 2, Czech Republic

**Keywords:** Ni-Ti alloy, self-propagating high-temperature synthesis, spark plasma sintering, aging, compressive test, hardness, shape memory

## Abstract

Ni-Ti alloys are considered to be very important shape memory alloys with a wide application area including, e.g., biomaterials, actuators, couplings, and components in automotive, aerospace, and robotics industries. In this study, the NiTi46 (wt.%) alloy was prepared by a combination of self-propagating high-temperature synthesis, milling, and spark plasma sintering consolidation at three various temperatures. The compacted samples were subsequently heat-treated at temperatures between 400 °C and 900 °C with the following quenching in water or slow cooling in a closed furnace. The influence of the consolidation temperature and regime of heat treatment on the microstructure, mechanical properties, and temperatures of phase transformation was evaluated. The results demonstrate the brittle behaviour of the samples directly after spark plasma sintering at all temperatures by the compressive test and no transformation temperatures at differential scanning calorimetry curves. The biggest improvement of mechanical properties, which was mainly a ductility enhancement, was achieved by heat treatment at 700 °C. Slow cooling has to be recommended in order to obtain the shape memory properties.

## 1. Introduction

The Ni-Ti alloys named NiTinol (derived from nickel, titanium, and laboratory of discovery-Naval Ordnance Laboratory) are well-known shape memory alloys with good mechanical properties and high corrosion resistance, which enables usage as implants, medical devices, and other applications as biomaterials [1]. The shape memory effects occur due to the reversible solid-state transformation between the high-temperature and low-temperature phases. The high-temperature phase is called austenite with a high-symmetry structure-ordered body-centered cubic phase B2 (CsCl). The low-temperature and low-symmetry phase is called martensite with monoclinic B19′ lattice. Reversible strains at about 8% of the initial length are enabled due to the reversible phase transformation as well [1]. To achieve the desired shape memory effects-transformation temperatures, it is necessary to be careful about a chemical composition. The transformation temperatures are very sensitive for the nickel-titanium ratio and an increase in the content of nickel by 0.1 at a percentage causes a change of the transformation temperature A_F_ (austenite finish) up to 10 °C [2,3]. This fact makes the production of the Ni-Ti alloys more difficult because titanium and, subsequently, NiTi alloys have high affinity to oxygen from atmosphere and carbon from the melting crucible during vacuum induction melting (VIM) [4,5].

Spark plasma sintering (SPS) is a modern consolidation process, which is suitable for various materials such as ceramics and metals including many intermetallic systems (e.g., Ni-Ti and Fe-Al alloys [6,7,8,9]). The high heating rate and shortness of the whole process enable the use of SPS for the consolidation of nanocrystalline materials as well [10,11,12]. The process is based on the sintering of powder under the simultaneous influence of high electric current (direct or pulsed) and uniaxial pressure. The Joule heat is generated by passing the current through the graphite punch and die and between the powder’s particles. The high heating rate was described as the route to reduce the amount of undesirable secondary phase such as the Ti_2_Ni by the self-propagating high-temperature synthesis (SHS) [13]. Thus, the SPS process was examined as a heating source for initiating the SHS reaction between nickel and titanium elemental powders in the previous paper. However, the SPS process seems to be inapplicable for the initiation of the SHS reaction because the strongest increase of the temperature occurs on the surface of the particles. The formed intermetallic layers act further as diffusion barriers and separate unreacted nickel from titanium [14]. Therefore, the pre-alloyed Ni-Ti powder after mechanical alloying is usually sintered by SPS. This process can produce the highly dense NiTi materials [7] or the porous structure depending on the addition of the space holder (e.g., NH_4_HCO_3_) [15,16].

Heat treatment of the Ni-Ti alloys has a crucial effect on the properties of the samples. The parameters of heat treatment influence the microstructure, internal stresses, precipitation, shape memory, and mechanical properties [17]. The Ni-Ti alloys undergo homogenizing treatment at the temperature of about 800–1050 °C up to several hours of duration, which is followed by water quenching to get a homogeneous microstructure without the Ni-rich precipitates [17,18,19,20,21,22]. The second step of heat treatment represents the aging treatment, which usually occurs between 300 °C and 800 °C [23,24]. The grade of aging (density and size of the Ni_4_Ti_3_ precipitates) depends on the temperature and time. The precipitation process starts with the metastable Ni_4_Ti_3_ phase, which is transformed into the metastable Ni_3_Ti_2_ phase and the stable Ni_3_Ti [25]. The precipitation process is accompanied by the hardness changes during aging due to formation, coarsening, and decomposition of the Ni_4_Ti_3_ and Ni_3_Ti_2_ phases. The addition of Al enhances the microstructural and hardness stability of Ni-rich Ni-Ti alloys until 500–600 °C [17,26]. The results of phase formation, stability, and transformation Ni_4_Ti_3_ → Ni_3_Ti_2_ → Ni_3_Ti during aging are presented by a time-temperature-transformation (T-T-T) diagram [17,25,27].

In this work, the NiTi46 (wt.%) alloy was processed by a combination of SHS, milling in a vibratory mill, and SPS consolidation at three temperatures to get fully dense materials. The prepared samples underwent the heat treatment in temperatures ranging from 400 to 900 °C. The characterization of samples was focused on phase composition, observing the transformation temperatures, and changing the mechanical properties depending on the heat treatment regime.

## 2. Materials and Methods

**Figure 1:**
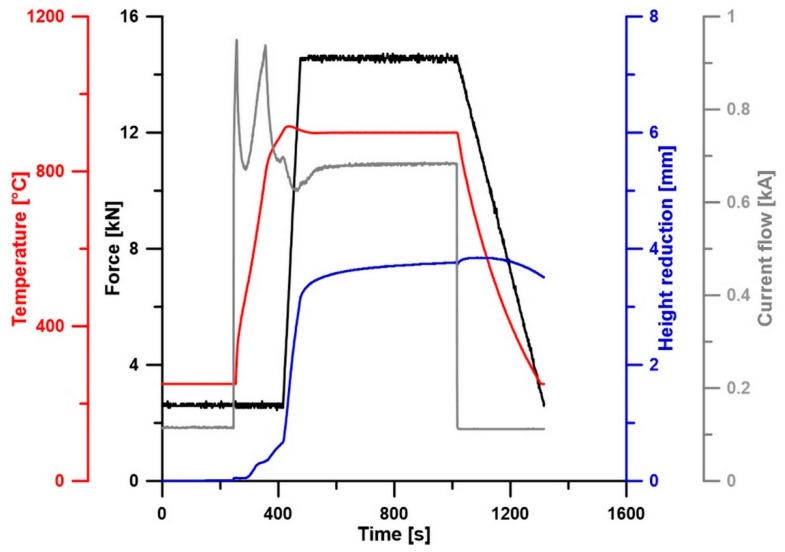
SPS parameters during consolidation at the temperature of 900 °C.

The metallic powders with the following particle sizes and purities were used as starting material for the NiTi46 (wt.%) alloy: nickel (particle size < 150 µm, 99.9 wt.% purity, Sigma-Aldrich, St. Louis, MO, USA) and titanium (particle size < 44 µm, 99.5 wt.% purity, STREM CHEMICALS, Newburyport, MA, USA). The powders were mixed manually corresponding to the chemical composition of the NiTi46 powder mixture, which was uniaxially compressed at room temperature to cylindrical green bodies of 12 mm in diameter at a pressure of 450 MPa for 5 min using LabTest 5.250SP1-VM universal loading machine (Labortech, Opava, Czech Republic). The SHS reaction of the pressed powder mixture was carried out in the fused silica ampoules evacuated to 10^−2^ Pa and sealed, which were placed in the preheated region to 1100 °C electric resistance furnace. The duration of the reaction was 20 min with the following cooling in air. The properties of the samples prepared this way were described in a previous paper [28]. The microstructure is composed of the two phases (NiTi austenite – cubic, Ti_2_Ni – cubic), hardness 276 HV10, area fraction of the Ti_2_Ni phase 11.7%, and transformation temperatures: A_S_ = 56 °C, A_F_ = 86 °C, and M_S_ = 21 °C. The SHS product was milled in a vibratory cylinder mill VM4 (OPS Přerov, Přerov, Czech Republic) in atmosphere with a duration of 7 min and the powder fraction with a particle size <355 µm was selected by sieving using Fritsch Analysette 3 device (FRITSCH GmbH, Germany). This pre-alloyed NiTi46 powder was consolidated by using the SPS method (FCT Systeme HP D 10, Frankenblicke, Germany) at three various temperatures (900, 1000, and 1100 °C) under the pressure of 50 MPa with a holding time of 10 min. The high heating rate was chosen as 300 °C/min at the beginning and the last 100 °C with the heating rate of 100 °C/min (for example, sintering temperature of 1000 °C: applied heating rate of 300 °C/min up to 900 °C, between the temperatures 900 and 1000 °C and the applied heating rate of 100 °C/min). The conditions of SPS consolidation as the temperature regime, compaction force, height reduction, and current flow are shown in Figure 1. 

The heat treatment in the temperature range from 400 °C to 900 °C was applied to the SPS-ed samples. The duration of heat treatment was 60 min. Two variants of cooling were used including a high cooling rate with quenching in water and a slow cooling rate, which was provided by cooling in the closed furnace (average cooling rate approximately 2.5 °C/min between 700 °C and 300 °C).

The metallographic samples were prepared by grinding and polishing and the microstructure was revealed by etching in Kroll’s reagent (5 mL HNO_3_, 10 mL HF, and 85 mL H_2_O). The microstructure was observed using scanning electron microscopes (SEM) equipped with the EDS (Energy Dispersive Spectroscopy) analyzers for identification of the chemical composition of the individual phases: VEGA 3 LMU (TESCAN, Brno, Czech Republic) equipped with the OXFORD Instruments X-max 20 mm^2^ SDD EDS analyzer (Oxford Instruments, HighWycombe, UK) and JEOL IT 500 HR 500 (JEOL, Tokyo, Japan). The phase composition was analyzed by the X-ray diffraction analysis (XRD) using a X’Pert Pro (PANalytical, Almelo, The Netherlands) X-ray diffractometer with CuKα radiation and a LynxEye XE detector (PANalytical, Almelo, The Netherlands). The mechanical properties of the samples were evaluated by measuring Vickers hardness with a load of 10 kg and compression tests (LabTest 5.250SP1-VM universal loading machine Labortech, Opava, Czech Republic) with a strain rate of 0.3 s^−1^ on samples measuring 3.3 mm × 3.3 mm × 5 mm. Compression tests were conducted in both the direction (longitudinal and perpendicular) to the direction of SPS. A longitudinal direction is parallel to compressive force during the SPS process. Differential scanning calorimetry (DSC) analysis of the prepared alloys was performed using Setaram DSC 131 (Setaram, Caluire, France) to determine the transformation temperatures in products. Measurements for determining temperatures austenite start (A_S_) and austenite finish (A_F_) were carried out in the temperature range of −20 °C to 200 °C at a heating rate of 10 °C/min and cooling from 200 °C to −5 °C for detecting the martensite start (M_S_) and martensite finish (M_F_) temperatures. 

The samples compacted by SPS at 900 °C and with following heat treatments at 600 and 700 °C for 1 h and slow cooling in the closed furnace were also investigated using transmission electron microscopy (JEOL JEM 2200FS, JEOL, Tokyo, Japan, accelerating voltage of 200 kV). Standard 3 mm samples prepared by a slow-speed diamond blade cutting were mechanically dimpled and ion polished in a Gatan PIPS 691 device (Pleasanton, CA, USA).

## 3. Results and Discussion

### 3.1. Microstructure, Phase Composition, and Phase Transformation

**Figure 2:**
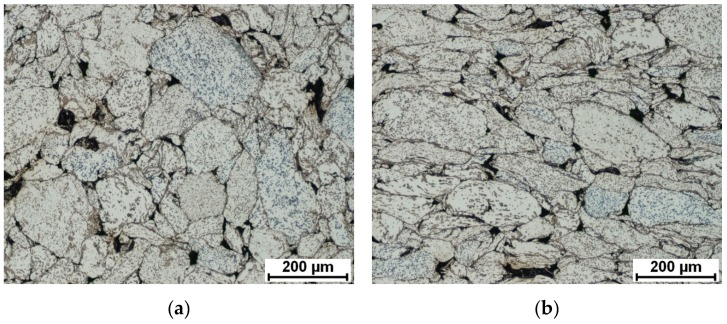

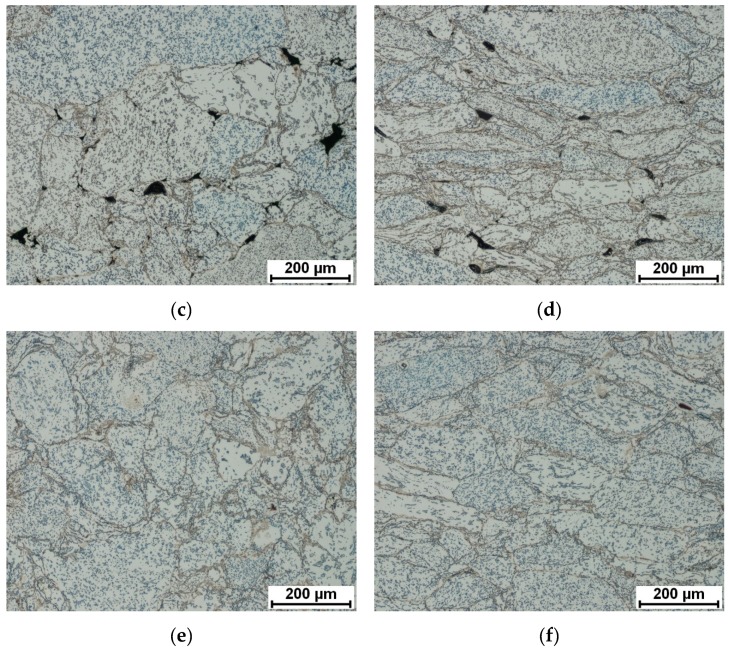
Microstructure and porosity of the NiTi46 alloy SPS consolidated at various temperatures: (**a**) 900 °C-perpendicular, (**b**) 900 °C-longitudinal, (**c**) 1000 °C-perpendicular, (**d**) 1000 °C-longitudinal, (**e**) 1100 °C-perpendicular, and (**f**) 1100 °C-longitudinal direction.

First, the influence of used sintering temperature on the quality of sintering individual particles was investigated by a porosity measurement. Since it is visible in Figure 2 and acquired by a light microscope, there are differences between samples compacted at various temperatures. The direction of observation plays an important role. The non-deformed grains were observed on the perpendicular cut (perpendicular to the direction of compression) whereas the elongated shape of grains after loading during SPS remained in the microstructure on the longitudinal cut (parallel to SPS compression). The highest values of porosity were determined at the samples sintered at 900 °C. The porosity was high at the perpendicular level and also at the longitudinal cut. The SPS temperature of 1000 °C was sufficient to the reduction of porosity in comparison to the temperature of 900 °C. Mainly in the longitudinal direction, the value of porosity decreased rapidly to a similar value, which was measured after sintering at 1100 °C. The values of porosity are compared in Table 1 and, based on the porosity measurement, it is clear that the higher temperature of the SPS process leads to superior sintering of individual particles.

The SPS temperature influences the phase composition and also mechanical properties. The effect of sintering temperature was investigated in the previous paper [29]. The area fraction of the undesirable Ti_2_Ni and Ni_3_Ti phases increased with an increasing sintering temperature. The high amount of the Ti_2_Ni phase was formed along the boundaries of the sintered particles. It is necessary to point out that, in the previous paper, pulse current flow through the sample (another SPS device) was used while, in this paper, the regime of direct current flow through the sample was applied and it is the reason for different results. In this case, the lower amounts of the Ti_2_Ni phase were measured generally and a growing trend with SPS temperature was not observed. The area fraction of the Ti_2_Ni phase increased slightly by SPS consolidation at 900 °C and 1000 °C, but, after SPS sintering at 1100 °C, the amount of the Ti_2_Ni phase was reduced to approximately 12%, which means a comparable value to result in samples prepared by the SHS method [29] (see Table 1).

**Figure 3:**
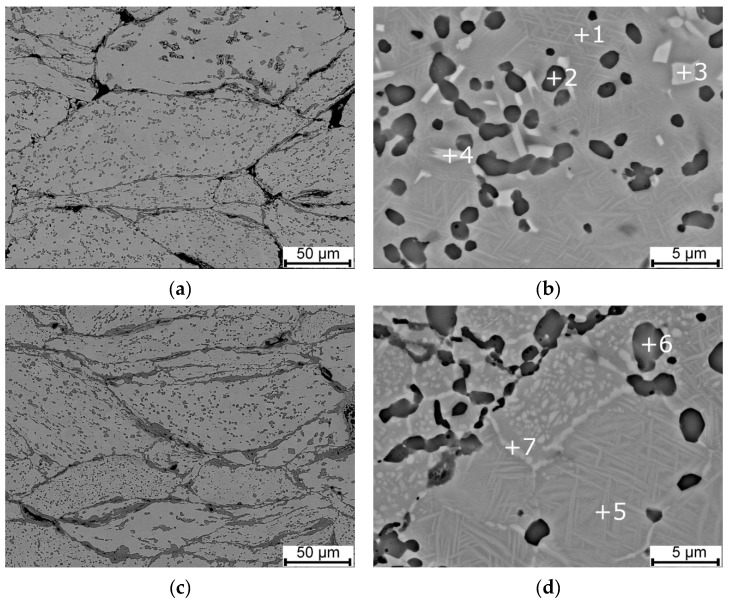

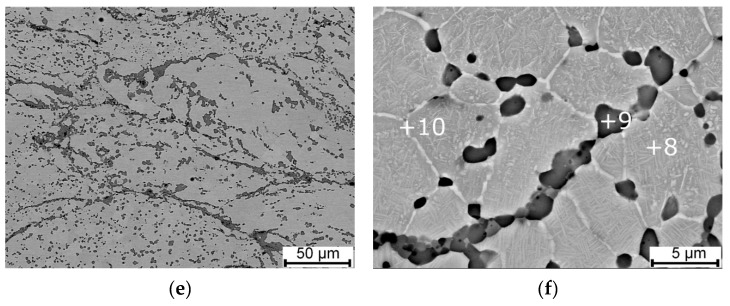
SEM micrographs of SPS-ed samples at various temperatures: (**a**) 900 °C, (**b**) 900 °C-detail of Ni-rich precipitates, (**c**) 1000 °C, (**d**) 1000 °C-detail of Ni-rich precipitates, (**e**) 1100 °C, and (**f**) 1100 °C-detail of Ni-rich precipitates and formed the Ni_3_Ti phase.

At all SPS temperatures, the SEM observation was performed. Improving the fusion of grain boundaries was confirmed with increasing sintering temperature. The NiTi phase matrix with the fine Ni-rich precipitates in all samples was commonly found within the Ti_2_Ni and Ni_3_Ti phases. The microstructures after SPS are shown in Figure 3. In Table 2, there are summarized chemical compositions of individual areas observed by SEM. A good agreement in chemical compositions of the NiTi and Ti_2_Ni phases to the binary Ni-Ti phase diagram was found out. The chemical composition of the area labelled 3 is close to the Ni_3_Ti phase. Chemical composition of areas 7 and 10 is approaching the chemical composition of the Ni_4_Ti_3_ phase.

**Figure 4:**
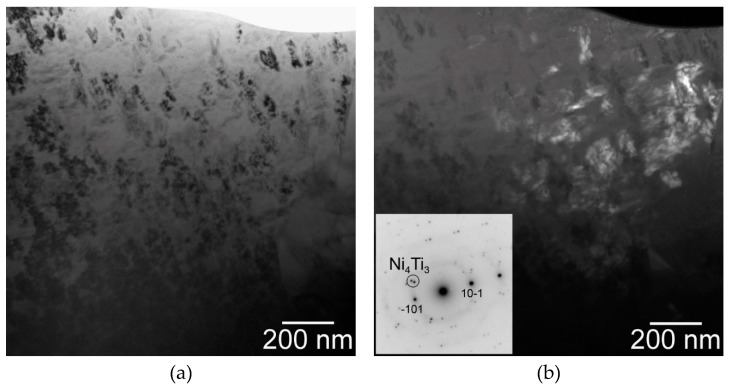

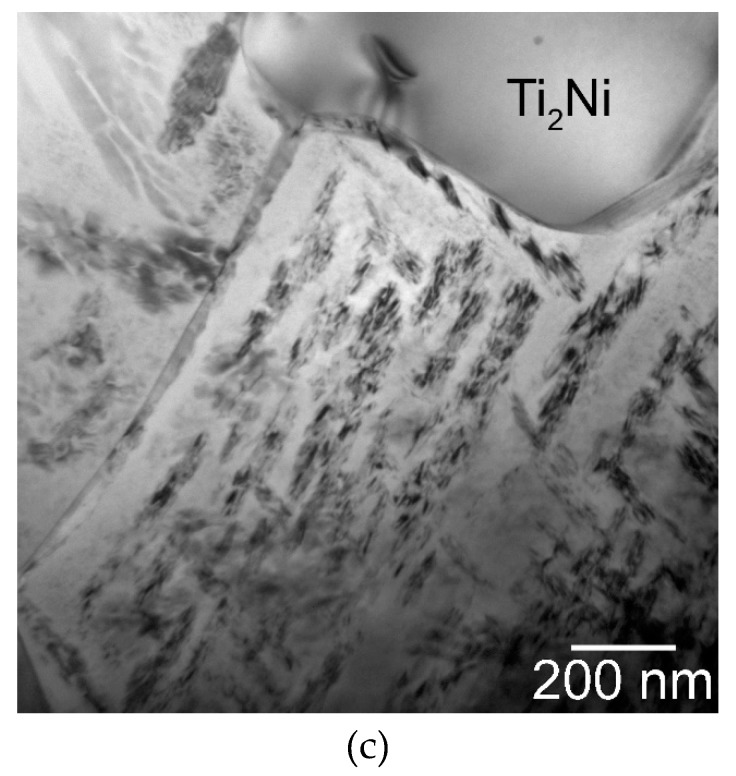
Ni_4_Ti_3_ phase in the matrix: (**a**) bright-field micrograph, g = 10-1, close to the [4,1,4] zone axis, (**b**) corresponding dark-field micrograph using Ni_4_Ti_3_ spot marked by a circle in the diffraction pattern in the inset, and (**c**) bright filed micrograph showing dark Ni_4_Ti_3_ particles in the matrix adjacent to a Ti_2_Ni particle.

A more detailed observation of the microstructure was performed using a transmission electron microscope (TEM). The main goal of this experiment is based on investigating the fine needle-like particles in the NiTi matrix. Figure 4 shows TEM micrographs of sample SPS-ed at the temperature of 900 °C. The NiTi and Ti_2_Ni phase were observed commonly with the Ni_4_Ti_3_ phase (determined by electron diffraction) in the NiTi matrix.

**Figure 5:**
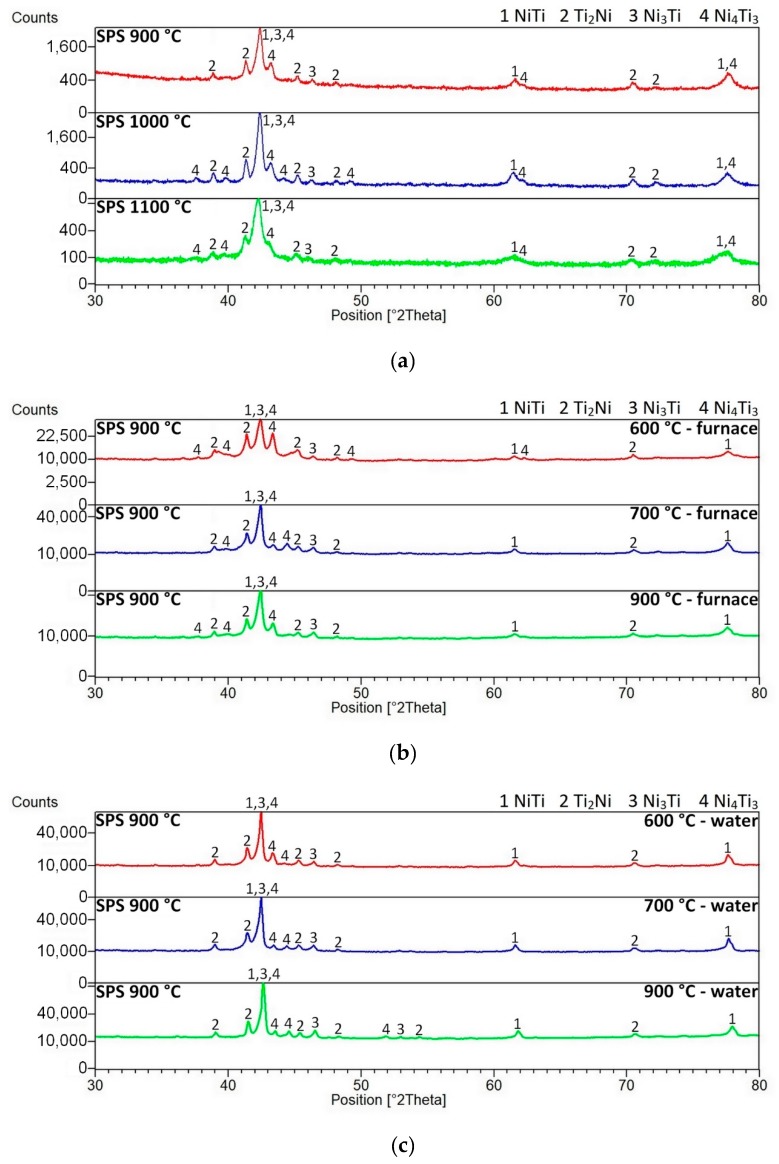
XRD patterns of the NiTi46 alloys: (**a**) spark plasma sintered at 900, 1000, and 1100 °C, (**b**) spark plasma sintered at 900 °C, heat-treated at 600–900 °C and slowly cooled, (**c**) spark plasma sintered at 900 °C, heat-treated at 600–900 °C, and cooled in water.

The effect of heat treatment on the microstructure and the phase composition was investigated at samples SPS-ed at the temperature of 900 °C. The phase compositions of SPS-ed samples were verified by XRD analysis. The diffraction lines were very similar through all SPS consolidation temperatures. The phase composition of SPS-ed samples consists of the NiTi phases (Cubic, Pm-3m), Ti_2_Ni phase (Cubic, Fd-3 m), Ni_3_Ti phase (Hexagonal, P63/mmc), and the Ni_4_Ti_3_ phase (Rhomboedral, R-3). XRD patterns are displayed. After heat treatment in the temperature range of 600–900 °C with a 1-h duration, there were no observed changes in the phase compositions of the samples (see Figure 5). This fact can seem to be strange in comparison with other studies. However, it is necessary to consider the initial state in individual studies (mostly after homogenization annealing, e.g., [17,27]). The initial state of these samples is after SPS consolidation at 900, 1000, and 1100 °C. The very high heating rate (approximately 300 °C/min) and a very high cooling rate (as shown in Figure 1) was applied during the SPS process, whereas the initial state of the Ni-Ti alloys is after homogenization or solution annealing for tens of minutes or several hours at temperatures at around 1000 °C [17,27,30]. Moreover, the transformations in the microstructure and phase composition occur in a bigger extent after heat treatment with longer duration since it is clear in this study [17]. The crystallite sizes of the NiTi phase after spark plasma sintering, which was determined by the means of the Sherrer’s formula, range from 20 to 47 nm. The crystallite sizes increased after heat treatment and the highest values of the crystallite sizes were determined at samples heat-treated at a temperature of 700 °C. The significant increase of the crystallite sizes of the NiTi phase is related likely to the recrystallization process, grain growth, or the order-disorder transformation around the temperature of 600–700 °C reported in References [31,32]. The values of the crystallite sizes of the NiTi phase depending on the regime of the heat treatment are stated in Table 3.

**Figure 6:**
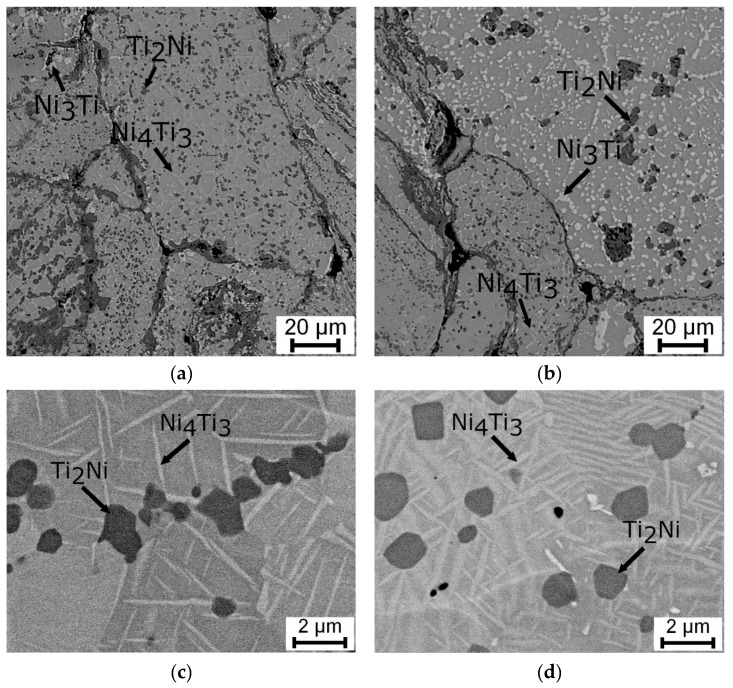
SEM microstructures of samples SPS-ed at 900 °C and heat-treated: (**a**,**c**) at 700 °C followed with slow cooling in the closed furnace, (**b**) at 700 °C and cooled in water, and (**d**) at 600 °C followed with slow cooling in the closed furnace.

**Figure 7:**
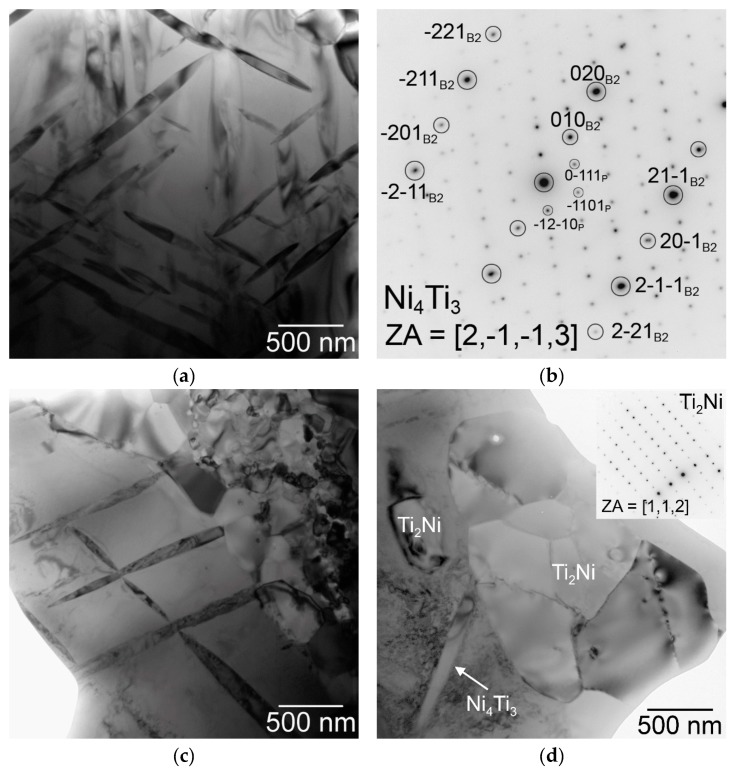
TEM observation of samples heat-treated at 600 °C (**a**,**b**) and at 700 °C (**c**,**d**) and slow cooled: (**a**) Ni_4_Ti_3_ precipitates with a typical lenticular cross-section, (**b**) corresponding diffraction pattern in the zone axis [2,-1-1,3] of the precipitate, which is coincident with the zone axis [1,0,2] of the B2 NiTi matrix, (**c**) Ni_4_Ti_3_ particles in the NiTi matrix adjacent to very fine grains of the Ti_2_Ni phase, (**d**) Ni_4_Ti_3_ particle adjacent to a coarser grain of the Ti_2_Ni phase. The inset shows a diffraction pattern of the Ti_2_Ni phase in the [1,1,2] zone axis.

From the point of view of the microstructure, the particles of the Ti_2_Ni, Ni_3_Ti phases and Ni_4_Ti_3_ needles in the NiTi matrix were observed in Figure 6. A higher amount of the Ni_3_Ti phase was formed in the sample heat-treated at 700 °C and cooled in water than in the samples with slow cooling in the closed furnace after heat treatment. The area fraction of the Ti_2_Ni phase after heat treatment was almost invariable with values between 13% to 16%. Detailed observation of the Ni_4_Ti_3_ and other phases after heat treatment at 600 °C and 700 °C was carried out by TEM again (see Figure 7).

**Figure 8:**
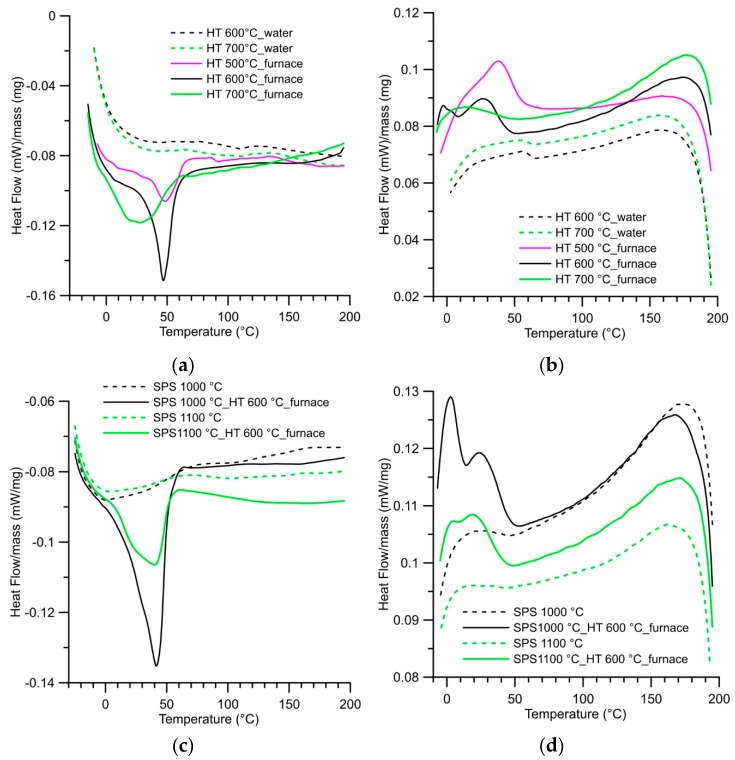
DSC heating and cooling curves: (**a**) Heating curves of samples SPS-ed at 900 °C and heat-treated between 500 and 700 °C. (**b**) Cooling curves of samples SPS-ed at 900 °C and heat-treated between 500 and 700 °C. (**c**) Heating curves of samples SPS-ed at 1000 and 1100 °C and SPS-ed at 1000 and 1100 °C with heat treatment at 600 °C. (**d**) Cooling curves of samples SPS-ed at 1000 and 1100 °C SPS-ed at 1000 and 1100 °C with heat treatment at 600 °C.

The phase transformation in the NiTi phase between austenite and the martensite structure was studied using differential scanning calorimetry. The straight lines were obtained for the samples as-SPS sintered at all temperatures and any phase transformation occurs in these samples. The change in the phase transformation behaviour was brought by heat treatment of the samples. When the samples were heat-treated, the temperature of heat treatment and way of cooling are important. Fast cooling in water did not cause the recovery of the phase transformation. Therefore, the microstructure observation and mechanical property investigation are focused on the samples cooled slowly in the closed furnace. The peaks on DSC curves were formed only at samples after heat treatment at temperatures of 600–700 °C, which were slow cooled in the closed furnace. The heating and cooling DSC curves of samples SPS-ed at 900 °C are shown in Figure 8.

### 3.2. Mechanical Properties

The increase of hardness with the increasing temperature of SPS is similar to the previous results [29]. However, an increase of hardness was assigned to the rising value of the Ti_2_Ni phase. It is contrary to the current study and to the decrease of the Ti_2_Ni phase amount after SPS at 1100 °C. There are two possible reasons. Firstly, the hardness increases with the quality of powder sintering at higher SPS temperature, which is visible in Figure 3. Secondly, the precipitation process of Ni-rich phases occurs during the SPS process while heat treatment at the temperatures of 900–1000 °C lead to improved hardness [17]. Values of hardness as SPS-ed samples are stated in Table 4.

**Figure 9:**
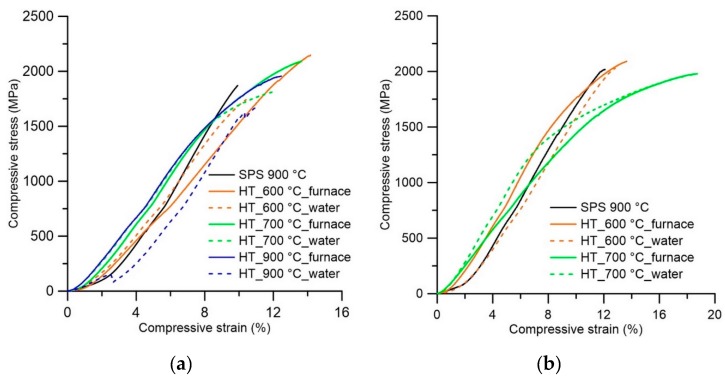

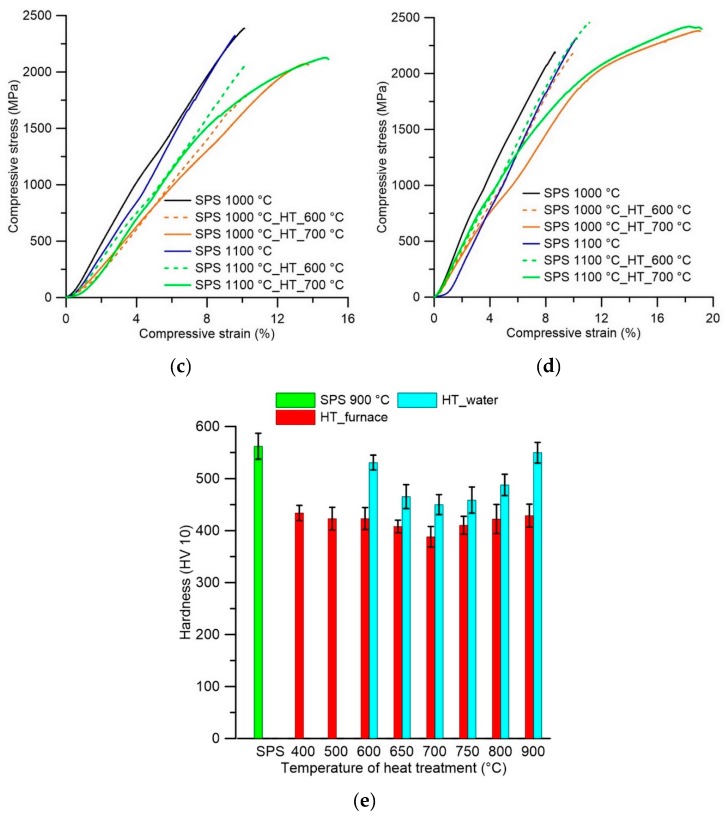
Compressive stress-strain curves and evolution of hardness: (**a**) Stress-strain curves of samples SPS-ed at 900 °C and heat-treated-perpendicular direction. (**b**) Stress-strain curves of samples SPS-ed at 900 °C and heat-treated-longitudinal direction. Stress-strain curves of samples SPS-ed at 1000 °C, 1100 °C, and heat-treated-perpendicular (**c**) and longitudinal (**d**) direction. (**e**) Evolution of hardness during heat treatment of sample SPS-ed at 900 °C.

The stress-strain behavior was analyzed in tandem with hardness and the same influence (increasing values of UCS) with an increasing temperature of the SPS process was observed. As visible in Figure 9, the samples after SPS reach high values of ultimate compressive strength (UCS) 1900–2300 MPa, but there are not the areas of plastic deformation on stress-strain curves and the samples fail in a brittle manner at a maximum load perpendicularly as well as in the longitudinal direction. The values of elongation at maximum force (Agt) were between 7.4–9.4% for all SPS temperatures and both directions. In the case of the compression test, the lower porosity of samples SPS-ed at higher temperatures is likely connected with increasing values of UCS.

After heat treatment with cooling in the closed furnace, the decrease of hardness was found and the lowest value was measured after heat treatment at the temperature of 700 °C. Currently, with the decrease of hardness, increased ductility and plasticity of samples was measured by a compressive test (Table 5). The evolution of hardness depending on the temperature of heat treatment is shown in Figure 9 for samples SPS sintered at 900 °C. In case of samples SPS sintered, a 1000 °C and 1100 °C, hardness after heat treatment at 600 °C dropped to values of 504 HV 10 and 509 HV 10. By the compressive test after heat treatment, the difference between annealing temperatures of 600 °C and 700 °C was observed. In case of annealing temperature of 600 °C, the similar values of Agt were measured at longitudinal and perpendicular directions between 9.5% and 10.5%, whereas, at an annealing temperature of 700 °C, the increase of Agt was found and the difference between longitudinal (16.3–19.1%) and perpendicular (9.3–13.6%) direction grew up. The UCS of the samples SPS-ed at 1000 °C and 1100 °C increased after heat treatment about 100 MPa in comparison with the state after SPS. Generally, the values of UCS and Agt increased with an increasing temperature of the SPS process.

The explanation of the rapid decrease of hardness could be caused due to removing the deformation strengthening from the milling of an SHS product. This type of decrease has to have the same scale for all samples. The dependence of hardness on the temperature of heat treatment is undeniable. Thus, the different processes and changes in microstructure must occur during heat treatment at various temperatures. However, these changes were not observed in phase composition and microstructure due to the short time of annealing during heat treatment. In this study [17], the longer time heat treatment was applied and the following changes in the microstructure occurred. The high UCS, hardness, and low ductility of samples were attributed to the precipitates of Ni_3_Ti_4_ and Ni_3_Ti_2_ phases, while, when the microstructure contains the Ni_3_Ti phase or combination of the Ni_3_Ti and Ni_3_Ti_2_ phases (corresponding to heat treatment between 600 and 800 °C while a longer time must be applied at a temperature of 600 °C), high mechanical properties and good ductility were obtained. Both studies [17,24] show similar evolution of the hardness values during heat treatment when compared to our results.

## 4. Conclusions

The fabrication process composed of Self-propagating High-temperature Synthesis (SHS) and Spark Plasma Sintering (SPS) was chosen to obtain a completely dense material. The highest temperature of SPS (1100 °C) led to the highest values of ultimate compressive strength without the formation of an excessive number of undesirable phases. The following heat treatment is necessary to obtain a material with a good combination of strength and ductility. The heat treatment leads to the disappearance of the deformation strengthening coming from milling in the vibration mill and to recover the phase transformation between the austenite and martensite structure of the NiTi phase, which was detected by differential scanning calorimetry (DSC). The heat treatment near the temperatures of 600–700 °C with slow cooling is recommended to obtain good ductility, strength, and probable shape memory properties.

## Figures and Tables

**Table 1:** Influence of SPS temperature on porosity of the sample and area fraction of the Ti_2_Ni phase in the microstructure.

Parameter	Direction	Spark Plasma Sintering Temperature		
		900 °C	1000 °C	1100 °C
Porosity (%)	Perpendicular	1.6 ± 0.09	0.7 ± 0.16	˂ 0.1
	Longitudinal	1.4 ± 0.09	0.1 ± 0.06	˂ 0.1
Area fraction of the Ti_2_Ni phase (%)		13.8 ± 2.41	17.0 ± 2.22	11.9 ± 1.22

**Table 2:** Chemical composition of individual areas measured by EDS analysis.

Area	Ni (wt.%)	Ti (wt.%)
1	54.4	45.6
2	38.0	62.0
3	72.4	27.6
4	68.6	31.4
5	55.5	44.5
6	38.0	62.0
7	58.7	41.3
8	55.2	44.8
9	37.6	62.4
10	59.1	40.9

**Table 3:** Crystallite sizes of the NiTi phase depending on the regime of heat treatment.

Sample	Crystallite Size (nm)
SPS 900 °C	47
SPS 1000 °C	34
SPS 1100 °C	20
SPS 900 °C-HT 600 °C-furnace	25
SPS 900 °C-HT 700 °C-furnace	122
SPS 900 °C-HT 900 °C-furnace	28
SPS 900 °C-HT 600 °C-water	84
SPS 900 °C-HT 700 °C-water	129
SPS 900 °C-HT 900 °C-water	57

**Table 4:** Summary of hardness and compressive stress-strain test of samples after spark plasma sintering at various temperatures. Longitudinal direction is parallel to the direction of compressive force by the SPS process.

SPS Temperature		900 °C	1000 °C	1100 °C
Hardness (HV 10)/Std. dev. (±)		562/25	596/20	624/23
Longitudinal	UCS (MPa)	1903	2116	2315
	Agt (%)	8.7	8.7	8.7
Perpendicular	UCS (MPa)	1953	2212	2243
	Agt (%)	7.4	9.4	8.6

**Table 5:** Summary of mechanical properties of heat-treated samples consolidated by the SPS process.

Sample	Heat Treatment Regime	Hardness (HV 10)/Std. dev. (±)	Direction	UCS (MPa)	Agt (%)
SPS 900 °C	600 °C-furnace	423/21	Perpendicular	2163	10.5
			Longitudinal	2089	11.6
SPS 900 °C	600 °C-water	531/14	Perpendicular	1508	7.6
			Longitudinal	1994	9.9
SPS 900 °C	700 °C-furnace	388/20	Perpendicular	1430	9.3
			Longitudinal	1907	16.3
SPS 900 °C	700 °C-water	450/19	Perpendicular	1566	9.3
			Longitudinal	1878	13.7
SPS 900 °C	900 °C-furnace	429/22	Perpendicular	1957	11.1
SPS 900 °C	900 °C-water	550/20	Perpendicular	1670	-
SPS 1000 °C	600 °C-furnace	504/21	Perpendicular	2089	9.5
			Longitudinal	2235	9.2
SPS 1000 °C	700 °C-furnace	444/14	Perpendicular	2099	12.5
			Longitudinal	2355	18.5
SPS 1100 °C	600 °C-furnace	509/22	Perpendicular	2295	10.0
			Longitudinal	2488	10.6
SPS 1100 °C	700 °C-furnace	448/10	Perpendicular	2163	13.6
			Longitudinal	2465	19.1
